# Supplementary material for: Programmed cell senescence in skeleton during late puberty
Source: Nat Commun. 2017 Nov 3;8:1312. doi: 10.1038/s41467-017-01509-0 (PMC5670205; doi:10.1038/s41467-017-01509-0)
Supplement: Supplementary file 1 — Supplementary Information [file 41467_2017_1509_MOESM1_ESM.pdf]

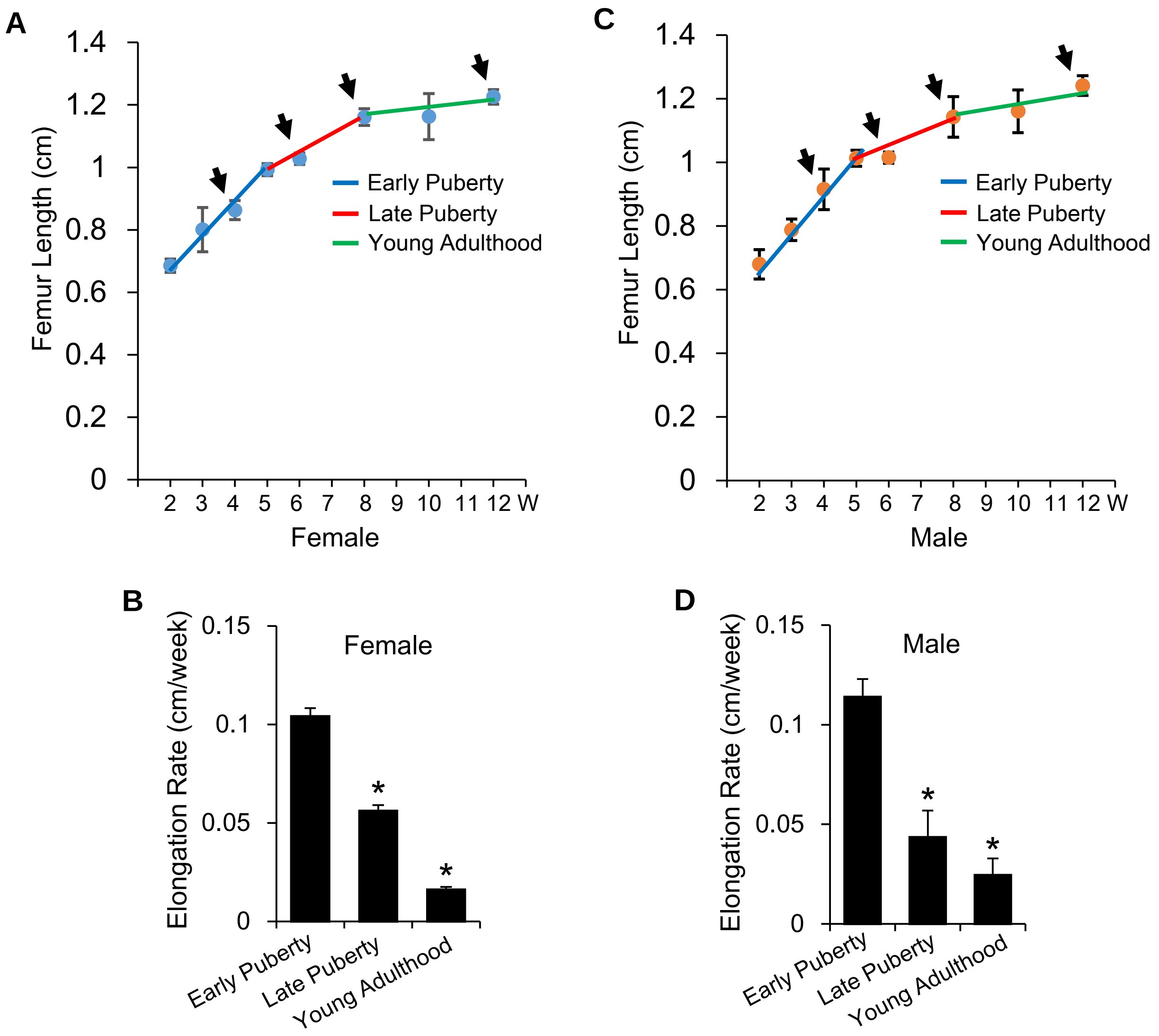

Supplementary Figure 1. Mouse Femur Length in Different Ages.
Femur lengths of 2-,3-,4-,5-,6-,8-,10-,12-week-old female (**A**) and male (**C**) *C57BL/6J* mice were measured using X-ray. Black arrows represent the designed time points for further analysis of cellular senescence in primary spongiosa of long bone; the blue lines represent early puberty period; the red lines represent late puberty period; the green lines represent young adulthood. The elongation rates of femora in early puberty, late puberty, and young adulthood were calculated from female (**B**) and male (**D**) *C57BL/6J* mice. Five mice per group. Data are represented as mean $\pm$ s.e.m. * $p<0.05$ versus early puberty as determined by ANOVA.

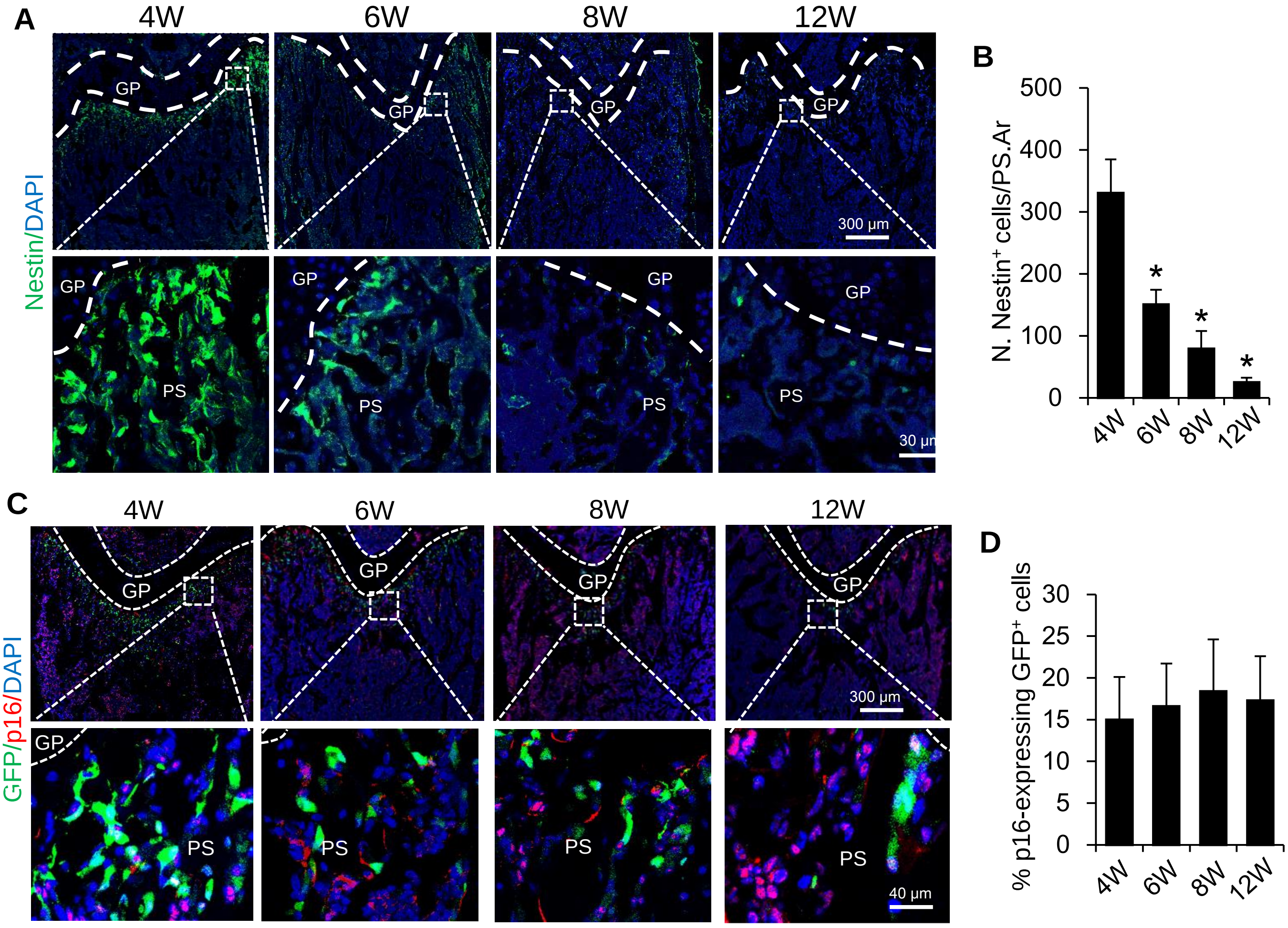

Supplementary Figure 2.

Nestin^{+} Cells in Primary Spongiosa of Long Bone Gradually Diminished During Late Puberty. Representative images of nestin immunofluorescence staining (**A,** nestin-positive signal is in green) and quantitative analysis (**B**) of nestin^{+} cells in primary spongiosa of femur sections from 4-, 6-, 8-, 12-week-old male *C57BL/6J* mice. Images in upper panels in (**A**) are lower power with boxes outlining the area of higher power in lower panels. DAPI stains nuclei blue. Numbers of nestin^{+} cells per mm^2 tissue area in primary spongiosa (N. Nestin^{+} cells/PS.Ar) (**B**). Double-immunofluorescence images of femoral metaphysis sections from 4, 6, 8, 12-week-old male *Nestin-GFP* mice using antibodies against GFP (green) and either p16 (red) (**C**). DAPI stains nuclei blue. Images in upper panels in (**C**) are lower power with boxes outlining the area of higher power in lower panels. Quantification of the percentage of GFP^{+} cells that express p16 (**D**) are shown. Five mice per group. Data are represented as mean $\pm$ s.e.m. GP, growth plate. PS, primary spongiosa. * $p<0.05$ versus 4-week-old mice group as determined by ANOVA.

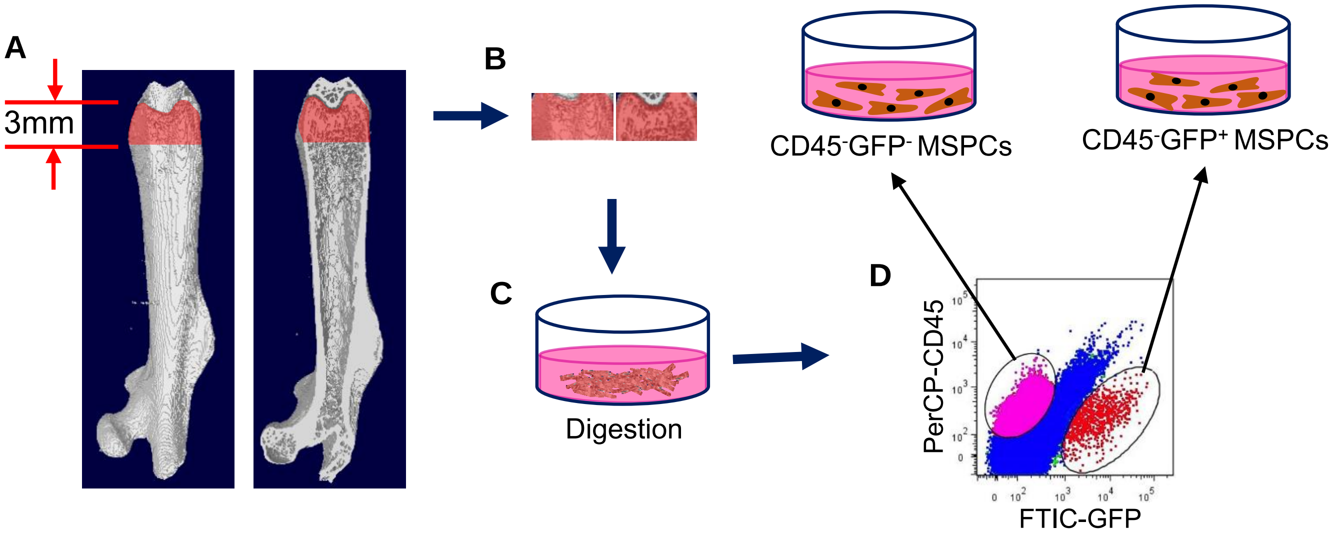

Supplementary Figure 3.

Steps to Isolate CD45$^-$GFP$^-$ and CD45$^-$GFP$^+$ Mesenchymal Stem/Progenitor Cells (MSPCs).
Femur of two month-old male *Nestin-GFP* mice were collected. The end of distal femur was removed at growth plate site, and the remaining bone were cut at 3 mm from the growth plate (**A**), and this piece of bone at the end was considered as metaphyseal region (**B**). To isolate cells from the metaphyses, the bone were then digested with a protease solution (2 mg/ml collagenase A and 2.5 mg/ml trypsin in PBS) for 20 min to remove the periosteum and periosteal progenitors (Step I). The bones were cut into small pieces and digested in the protease solution for another 1 h (**C**, Step II). Cells within the supernatant were collected for flow cytometry. CD45$^-$GFP$^+$ cells were then sorted according to side scatter and GFP-FITC fluorescence at > 10^3 log Fl-1 (GFP-FITC) fluorescence after negative selection of leucocyte common antigen CD45 at < 10^3 log Fl-3 (CD45-PerCP) fluorescence. CD45$^-$GFP$^-$ cells were then sorted according to side scatter and GFP-FITC fluorescence at < 10^3 log Fl-1 (GFP-FITC) fluorescence after negative selection of leucocyte common antigen CD45 at < 10^3 log Fl-3 (CD45-PerCP) fluorescence. The sorted cells were plated for expending (**D**). The adherent cells were considered as CD45$^-$GFP$^+$ and CD45$^-$GFP$^-$ MSPCs, which were used for further culture and analysis.

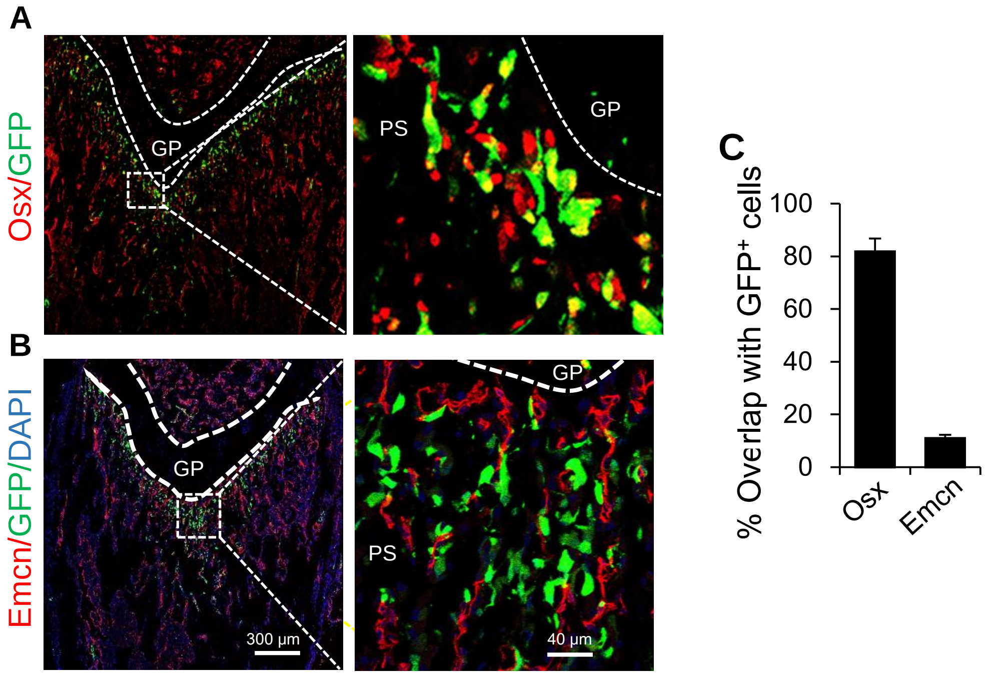

Supplementary Figure 4.

Nestin-GFP Labels Both Osteoprogenitor Cells and Vascular Endothelial Cells.

Double-immunofluorescence staining of metaphysis of femur sections from 4-week-old male *Nestin-GFP* mice was performed using antibodies against GFP (green) and either osterix (Osx) (red) (**A**) or endomucin (Emcn) (red) (**B**), respectively. Images in the left panes are lower power with boxes outlining the area of higher power in the right panels. DAPI stains nuclei blue. Quantification of the percentages of GFP^+ cells that express Osx or Emcn, respectively (**C**). Five mice per group. Data are represented as mean $\pm$ s.e.m. GP, growth plate. PS, primary spongiosa.

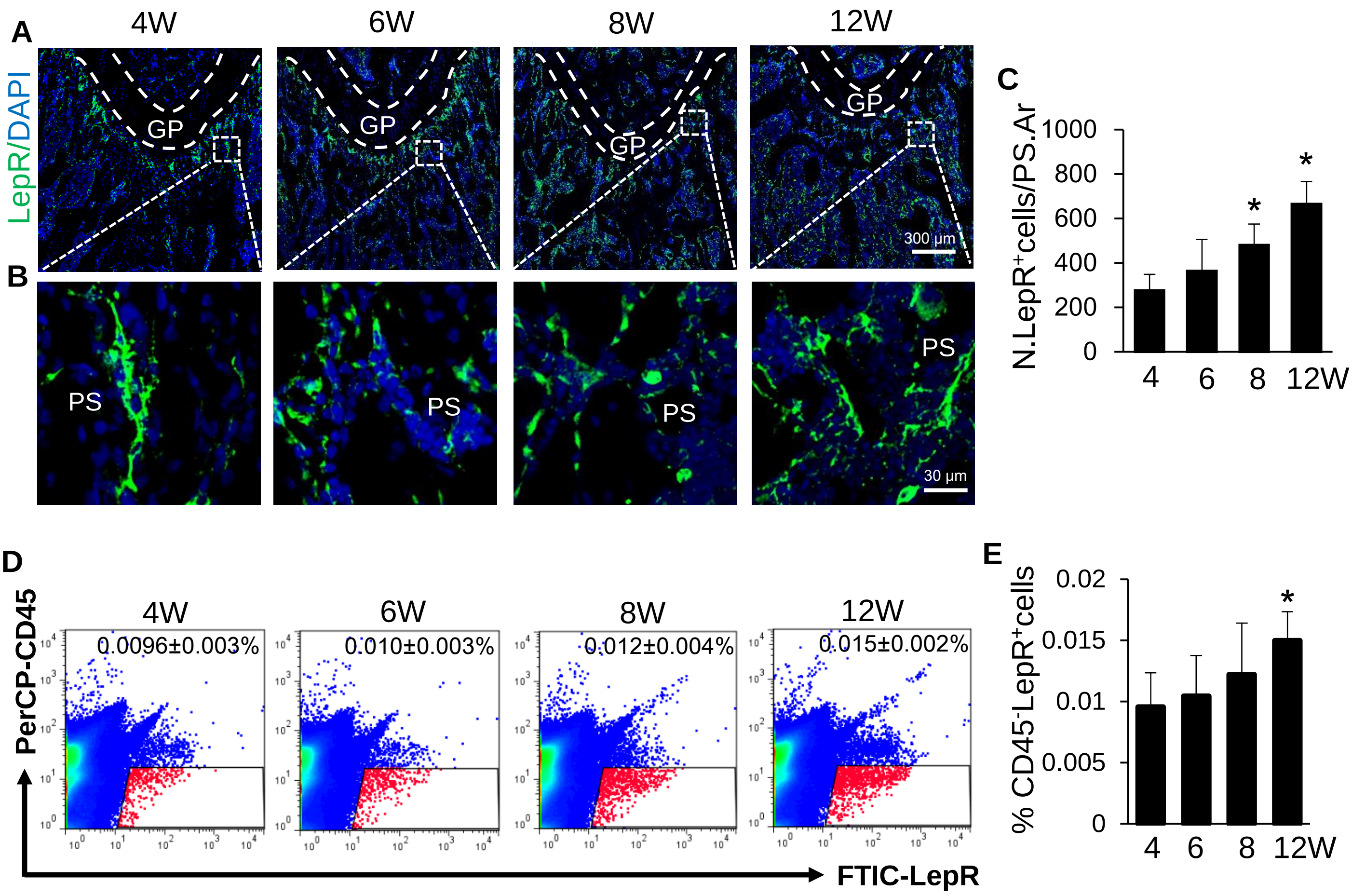

Supplementary Figure 5.

LepR⁺ Cells in Primary Spongiosa of Long Bone Gradually Increase During Late Puberty. Representative images of immunofluorescence staining (**A, B**) and quantitative analysis (**C**) of leptin receptor (LepR)$^{+}$ cells in primary spongiosa of femur sections from 4, 6, 8, 12-week-old male *C57BL/6J* mice. Images in (**A**) are lower power with boxes outlining the area of higher power in (**B**). Numbers of LepR^{+} cells per mm^{2} tissue area in primary spongiosa (N. LepR^{+} cells/PS.Ar) (**C**). Representative images of the flow cytometry analysis (**D**) and the percentage (**E**) of the LepR^{+} cells isolated from the metaphysis region. CD45^{-}LepR^{+} cells were then analyzed according to side scatter and LepR-FITC fluorescence at > 10^{1} log Fl-1 (LepR-FITC) fluorescence after negative selection of leucocyte common antigen CD45 at < 10^{1} log Fl-3 (CD45-PerCP) fluorescence. Six mice per group. Data are represented as mean ± s.e.m. GP, growth plate. PS, primary spongiosa. * $p<0.05$ vs. 4-week-old mice group as determined by ANOVA.

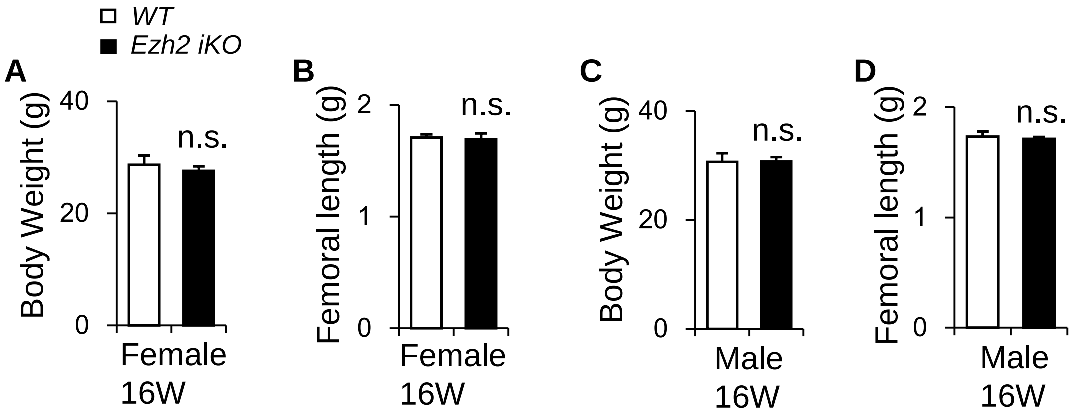

Supplementary Figure 6.

Mice with deletion of Ezh2 in Nestin⁺ Cells Have Unchanged Body Weight and Femoral Length.

Three-week-old female (**A, B**) and male (**C, D**) *Nestin-Cre*^{ERT2}*::Ezh2* $^{flox/flox}$ (*Ezh2 iKO*) mice and *Nestin-Cre*^{ERT2} mice (*WT*) were injected with 3 doses of tamoxifen (every other day). The mice were humanely killed at 16 weeks of age. Body weight (**A, C**) and femora length (**B, D**) of the mice were measured. Six mice per group. Data are represented as mean ± s.e.m. n.s, not significant as determined by Student's *t*-tests.

Supplementary Table 1. Primers used for ChIP-PCR

Primers	Forward	Reverse	TSS	Ref
p15^{INK4b}-1	CCGCCTAGAGATCGAACTAGCC	CGCTTTTGCAATTGACTGAC	-595~ -481	1
p15^{INK4b}-2	CACCGAAGCTACTGGGTCTC	CTGTGGCAGAAATGGTCCTT	-107~ -15	1
p15^{INK4b}-3	ATGTTCTAAGAGGCTTTGTTTCCA	CATTTGTGCATAGGAGATCAGG	+1542-+1623	1
p16^{INK4a}-1	TCCGATCCTTTAGCGCTGTT	CCCGGACTACAGAAGAGATG	-252 ~ -184	2
p16^{INK4a}-2	AGGGGTGTTCAATTCATGCTAT	ACACTCTGCTCCTGACCTGG	-130 ~ -50	2
p16^{INK4a}-3	GGAGCCACCCATTAAACTAACT	CAAAAATAAGACACTGAAAACTCG	+341 ~ +411	2
p19^{ARF}-1	AGGTGCCTCAACGCCGAAG	CTGGTCCAGGATTCCGGTGCGG	-12503 ~-12222	3
p19^{ARF}-2	ATGGGCAACGTTCACGTAGCAGC	AGCGGTACACAAAGACCACCCA	+5135~+5417	3
p21^{CIP1}	CACAGTTGGTCAGGGACAGA	CAGGACCAACCCACTCCTT	-4933~-4694	4
p27^{KIP1}	CTGGCTCTGCTCCATTTGAC	GGCTCCCGTTAGACACTCTC	-163~+31	4
Runx2	GGGGGAAGCCACAGTGGTA	AAAATAGGAGTTTTAAAGCT	-137~-22	5
Osteocalcin	GAGGACATTACTGAACAC	CAGTGGGTCAAACCCAAA	-505~-153	6
Gapdh	CCACCCAGAAGACTGTGGAT	GGATGCAGGGATGATGTTCT	+3767~+3850	1

Supplementary References

1. He J, Kallin EM, Tsukada Y, Zhang Y. The H3K36 demethylase Jhdm1b/Kdm2b regulates cell proliferation and senescence through p15(Ink4b). *Nat Struct Mol Biol* **15**, 1169-1175 (2008).

2. Kaneda A*, et al.* Activation of Bmp2-Smad1 signal and its regulation by coordinated alteration of H3K27 trimethylation in Ras-induced senescence. *PLoS Genet* **7**, e1002359 (2011).

3. Agherbi H, Gaussmann-Wenger A, Verthuy C, Chasson L, Serrano M, Djabali M. Polycomb mediated epigenetic silencing and replication timing at the INK4a/ARF locus during senescence. *PloS one* **4**, e5622 (2009).

4. Li J, Han S, Cousin W, Conboy IM. Age-specific functional epigenetic changes in p21 and p16 in injury-activated satellite cells. *Stem cells* **33**, 951-961 (2015).

5. Tamiya H*, et al.* Analysis of the Runx2 promoter in osseous and non-osseous cells and identification of HIF2A as a potent transcription activator. *Gene* **416**, 53-60 (2008).

6. Lee KN*, et al.* Orphan Nuclear Receptor Chicken Ovalbumin Upstream Promoter-Transcription Factor II (COUP-TFII) Protein Negatively Regulates Bone Morphogenetic Protein 2-induced Osteoblast Differentiation through Suppressing Runt-related Gene 2 (Runx2) Activity. *Journal of Biological Chemistry* **287**, 18888-18899 (2012).

Supplementary Table 2. Primers used for quantitative real-time PCR

**Primers**	**Forward**	**Reverse**
p16^{INK4a}	GAAAGAGTTCGGGGCGTTG	GAGAGCCATCTGGAGCAGCAT
p21^{CIP1}	AGAAGGTACTTACGGTGTGGT	GAGAGATTTCCCGAATTGCAGT
p53	ATCGCCTTCGACATCATCGC	CCCCATGCGTACTCCATGAG
Ki67	ACCGTGGAGTAGTTTATCTGGG	TGTTTCCAGTCCGCTTACTTCT
Ezh1	CCAGACTGCCAGAATCGCTTT	CAGGTGCTTTTTGAGGCCA
Ezh2	AGTGACTTGGATTTTCCAGCAC	AATTCTGTTGTAAGGGCGACC
Utx	CGGGCGGACAAAAGAAGAAC	CATAGACTTGCATCAGATCCTCC
